# Periscope of Dermatology

**Published:** 1898-11-12

**Authors:** 


### PERISCOPE OF DERMATOLOGY.

(*Continued from page* 100.)

**Eczema.**—Jamieson[1] describes a chronic form of eczema affecting the palms and soles only, and causing often a good deal of limitation of movement in the hands, due to drying and thickening of the epidermis. He mentions that the disease is more common in women than in men, and often is associated with the menopause. Other causes are gout, worry, and local irritation. The chief diseases resembling it are syphilis and arsenical keratosis. For treatment he recommends Unna's method by oxidised pyrogallic acid. The part is prepared by repeated poultices of starch, with which is incorporated some boric acid; these are changed every four or six hours. In the course of four or five days the palms become soft, and the following ointment is thoroughly rubbed in: ℞. Acidi pyrogallici oxidati, grs. 5 to 30, lanolini. oz. ½, ol. amydalae. aq. destillatae, aa. 2 drachms, M. fiat ung. He considers the action of this ointment is quite remarkable in restoring normal keratinisation, and states that the cures have been permanent. After a cure has been effected, only the blandest soaps should be used, and the feet should be rubbed daily with a loofah.

Brooke[2] believes that the initial lesion of eczema is usually chronic, and that vesicles are usually only a secondary manifestation. He does not, however, think that every kind of eczema is parasitic in origin, and amongst the non-parasitic varieties he draws special attention to "trade eczema," in which the poisonous substances act as irritants, destroying the epithelium, and producing an eczematous eruption. These cases, after lasting a long time, frequently take on a gyrate or circinate outline, which is so characteristic of parasites, and such lesions require the application of anti-parasitic remedies for their cure. He states that acute eczema of the hands is usually due to heat, friction, foreign bodies, inoculation with pus, cocci, or other micro-organism. Finally he refers to the form of palmar eczema described by Jamieson, which he has found exclusively in old people, especially in women. He considers that vaso-motor disturbance of the centres supplying the palms and soles is largely responsible for its development. He recommends the application of compresses saturated with 1 or 2 per cent. watery solution of resorcin, after which Unna's oxidised pyrogallol treatment should be used. The most important factor in preventing relapse is the absolute obliteration of the last remnants of the eczematous infiltration.

Pendred[3] records a case of keratosis palmaris in a woman aged 36, who had suffered from the complaint since infancy. From time to time the horny layer was shed in flakes, leaving a raw, bleeding surface and deep fissures. The interesting point about the case was that the disease had been handed down in unbroken succession for a period of 150 years, through five generations. It had been transmitted mainly, but not entirely, through the female line; but the offspring of those who had escaped were also unaffected.

Nelson[4] recommends one of the following methods of treatment in subacute or chronic eczema of the hand: (1) The application of a 20 per cent. salicylic acid plaster. (2) The constant application of a pancreatic emulsion or glycerol of papoid. (3) The continuous employment of a salicylic and ichthyol ointment, 10 per cent. of each. (4) Painting the part with pure liq. carbonis detergens each night and morning, lanoline being applied afterwards. By these methods not only is the hypertrophy and infiltration removed, but at the same time the disease itself is cured. After getting rid of the thickened epidermis, an ointment, paste, gelatine, varnish, or plaster containing salicylic acid and ichthyol, can be used, the strength of which will vary according to the degree of acuteness or chronicity of the lesion. The tars are also serviceable.

Hutchinson[5] describes a case of nummular eczema occurring in a man aged 28 which had existed for six months. The disease was perfectly symmetrical and general, and consisted of abruptly margined nummular patches, which varied in size from a sixpence to half a crown. The extensor surfaces were more affected than the flexor, and on the backs of the elbows the disease was diffuse. He considered the disease was constitutional in origin, and allied it with psoriasis; he, therefore, did not recommend any local treatment, but advised the internal administration of a sixteenth to an eighth of a grain of tartarised antimony three times a day.

[1] Brit. Jour. of Derm., April, 1898, p. 151, L.W. [2] Med. Chron., April, 1898, p. 26. [3] Epit. Brit. Med. Jour., April 30, 1898, p. 1,132. [4] Therapeutist, July 11, 1898, p. 167. [5] Med. Press, April 29, 1898, p. 406.

